# Feasibility and acceptability of a novel, computerized screening and brief intervention (SBI) for alcohol and sweetened beverage use in pregnancy

**DOI:** 10.1186/s12884-014-0379-x

**Published:** 2014-11-25

**Authors:** Madhabika B Nayak, Rachael A Korcha, Lee A Kaskustas, Lyndsay A Avalos

**Affiliations:** Alcohol Research Group-Public Health Institute (ARG-PHI), 6475 Christie Avenue, Suite 400, Emeryville, CA USA; ARG-PHI & School of Public Health, University of California, Berkeley, USA; Kaiser Permanente Northern California, Division of Research, Oakland, CA USA

**Keywords:** Screening and brief intervention, Alcohol, Sugar sweetened beverages

## Abstract

**Background:**

Recommended screening and brief intervention (SBI) for alcohol use during pregnancy is impeded by high patient loads and limited resources in public health settings. We evaluated the feasibility, acceptability and validity of a new self-administered, single-session, bilingual, computerized Screening and Brief Intervention (SBI) program for alcohol and sugar sweetened beverage (SSB) use in pregnancy.

**Methods:**

We developed and tested the computerized SBI program at a public health clinic with 290 pregnant women. Feasibility, acceptability, and validity measures were included in the program which had several modules, including those on demographics, health and beverage use. Time to complete the program and user experience items were used to determine program feasibility and acceptability. Validity analyses compared proportions of prenatal alcohol use identified by the program versus in-person screening by clinic staff.

**Results:**

Most program users (87%, n = 251) completed the entire program; 91% (n = 263) completed the key screening and brief intervention modules. Most users also completed the program in ten to fifteen minutes. Program users reported that the program was easy to use (97%), they learned something new (88%), and that they would share what they learned with others (83%) and with their doctors or clinic staff (76%). Program acceptability did not differ by age, education, or type of beverage intervention received. The program identified alcohol use in pregnancy among 21% of users, a higher rate than the 13% (p < .01) found via screening by clinic staff.

**Conclusions:**

Computerized Screening and Brief Intervention for alcohol and SSB use in public health clinics is feasible and acceptable to English and Spanish speaking pregnant women and can efficiently identify prenatal alcohol use.

## Background

Competing priorities of high patient loads, multiple patient health issues, and resource limitations in public health clinics impede implementation of evidence-based screening and brief interventions (SBI) for alcohol use during pregnancy [1]. Computerized SBI offers a potential solution to ensure that pregnant women in busy prenatal clinics receive these services [2]. Computerized SBI can innovatively increase access to care with minimal costs and staff resources and has several other advantages. These include portability and ensuring intervention fidelity as the SBI is delivered in a consistent and standard way. Use of computerized SBI is supported by the significant body of literature in college drinkers [3] and primary care populations [4] and, thus, recommended for the reduction of alcohol consumption and related problems by federal health agencies [5].

In 2000, Kaskutas and colleagues developed Early Start Plus (ESP), a novel computerized intervention for pregnant women. The ESP intervention incorporated detailed drink size assessment and feedback into Early Start (ES), a prevention program for pregnant women enrolled in a health maintenance organization [6,7]. A unique element of the ESP intervention is the assessment of drink size. Many drinkers, including pregnant women, and particularly heavy drinkers, pour and consume drinks that are larger than standard [8,9]. Because pregnant, heavier drinkers under-estimate the size of their drinks and under-report how much they drink [10,11], intervention with childbearing age women should include drink size assessment to better measure drinking. For women who are unable to stop drinking, drink size information can be used to encourage reduced drinking and, thereby, facilitate harm reduction.

Randomized control trial data showed that ES and ESP recipients had better neonatal and maternal outcomes, i.e., higher birth weight and lower rates of preterm labor respectively, than controls [7]. However, because ESP needs a health care provider to administer it, it is not a good match for today’s financially-constrained health care environment.

More recently, Tenkku and colleagues [12] developed a four-module intervention that was delivered online or by mail. A trial with 458 women showed that both versions reduced heavy drinking and increased contraceptive use in women of childbearing age. Web-based SBI has also been used with 150 women in a public health clinic [13] and was found to have high acceptability (96% women users found the program useful and interesting). Reduced drinking was reported in 75% of women who used the program but the 2 month-follow up drinking rates did not differ between those who did and did not use the program. Since follow up outcomes were assessed via telephone, the researchers hypothesized that social desirability issues or fear of losing WIC benefits may have biased results by reducing reporting of drinking by the control group.

Thus, existing computerized SBI programs have limited appeal as they require clinical staff [6,7], multiple sessions [12], or internet access [13] and are only available in English. We addressed these limitations by converting the single session ESP program into a stand-alone, self-administered, computerized SBI that does not require internet access. Because Hispanic women represent increasing proportions of the women in public prenatal care in the United States due to higher birth rates [14], we developed the program in both English and Spanish.

Another innovation of our new computerized SBI is the inclusion of sugar sweetened beverages (SSB). While reducing prenatal alcohol use has been a longstanding priority [15], SSB consumption has only recently been identified as a public health problem [16]. There is a lack of published data on the prevalence of SSB use among pregnant women. However, SSB use is highest among those at greatest risk for obesity and diabetes, both of which increase the risk of obstetric complications and adverse pregnancy outcomes [17]. Integrating SSBs into alcohol SBI programs offers an opportunity to address consumption of SSBs, particularly for pregnant women who do not drink alcohol. Hispanic women are more likely to consume SSBs than other ethnicities [18].

The objective of our study was to examine the feasibility, acceptability and validity of our self-administered, single-session, bilingual (Spanish and English) computerized SBI for alcohol and SSB use during pregnancy as implemented in a public health clinic.

## Methods

### Study design

Our study involved a test implementation of the computerized SBI program and therefore used an observational design. Expert review was used to ensure relevance and 5^th^ grade readability of all program materials. The computerized SBI program was pilot tested at a local public health clinic. Implementation was preceded by 4 weeks of intensive beta testing, specifically testing for software programming errors or failures, by the first author and 2 research assistants, and administration to 30 randomly selected women in the clinic.

All study protocol and procedures were reviewed and approved by the Institutional Review Boards of the Public Health Institute (PHI) and the Contra Costa Medical Research Center.

The SBI program was administered using an ATM-like kiosk with a 19-inch touch-screen monitor. The kiosk was placed in the waiting area of the clinic in order to increase its visibility and facilitate use. All pregnant women attending the clinic for the first time were provided a brightly coloured handout about the study with the clinic paperwork. Posters about the study were also posted conspicuously on the clinic walls. No other active recruitment was used for the study, i.e., no research personnel were present at the clinic to recruit participants and no incentives were provided for participation. Participants provided consent electronically by selecting “I agree” on the pertinent screen of the computerized program. Consent screens included contact information for the principal investigator (the first author) and the PHI IRB chair. Printed copies of this contact information were also visibly placed near the kiosk and available with clinic staff.

### Study setting and participants

Participants were recruited at a Women Infant and Children’s (WIC) clinic in northern California from June 2012 through January 2013. The federally funded WIC program serves a large portion of childbearing age women across the United States, mostly low-income and of minority status (54% Hispanic, see Table 1). In California, WIC serves 62% of all infants born in the state. At the study clinic, attendees are typically young (67% under 30 years), not married (66%), of non-white ethnicity (95%), and have high school or less education (60%). Roughly 140 new pregnant women enroll in the WIC program at the clinic each month.

**Table 1:** **Demographics of WIC clinic attendees, computerized SBI program users and users reporting alcohol use during pregnancy**

	**WIC attendees (averaged across month)**	**All program users**	**Users who used alcohol during pregnancy**	**Past 30 day sweetened beverage use**
	**(n = 765 to 840)**	**(n = 290)**	**(n = 60)**	**(n = 253)**
**Age**	%	%	%	%
Under 21	16	15	12	16
21-29	51	52	53	53
30-39	29	31	32	29
40 or more	3	2	3	2
Marital status*				
Married	34	31	25	30
Living with partner	-	29	18	30
Single/divorced/separated	-	40	57	40
**Race/Ethnicity**				
White	5	5	10	5
Hispanic	63	62	38	62
Black	20	16	30	17
Other^^	1	9	10	9
More than one race	2	8	12	8
Missing	9	-	-	--
**Preferred language**				
English	64	60	80***	59
Spanish	35	40	20	41
**Education**				
Less than High School	29	24	15*	23
Completed high school	31	37	33	37
Some college	12	28	45	29
College or more	-	11	7	11
Missing	28	-	-	--

*p < 0.05; ***p < .001.

Reported alcohol use in the past 30 days or in the 12 months before pregnancy.

^^^^Includes Asian, Pacific-Islander/Hawaiian, Alaska Native/American Indian, middle eastern and other”.

### Materials and measures

#### Overview of the computerized SBI Program

**Figure 1:**
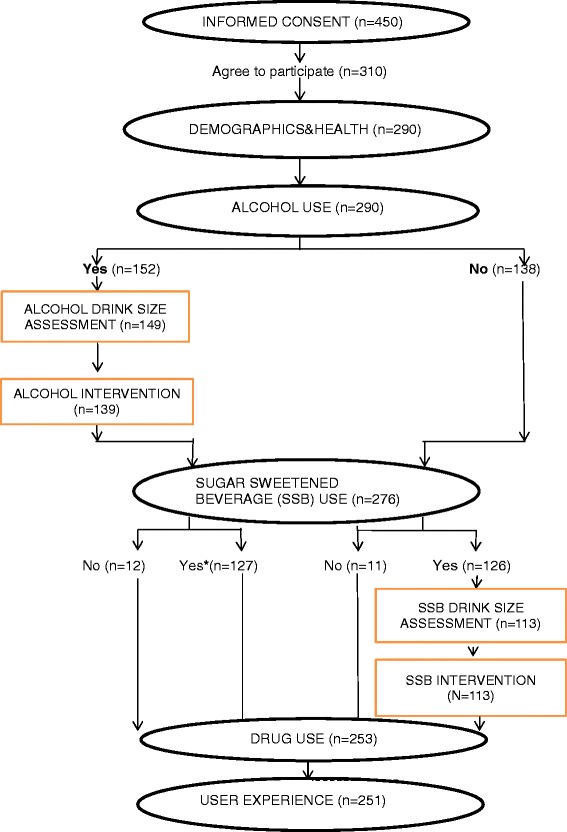
Kiosk-based computerized screening and brief intervention program modules and number of users completing each module.*To minimize user burden, alcohol users did not complete the SSB drink size assessment and intervention modules.

The program was developed as a self-administered, computerized, bilingual (English and Spanish) SBI. The screening assessed alcohol and sugar sweetened beverages (SSB) consumption and provided a brief intervention for women reporting consumption of these beverages (see Figure 1). The first module obtained informed consent and instructed program users on how to use the touch-screen program. The second module included questions assessing basic demographic and health, including age, marital status, education, ethnicity, number of visits to the clinic, gestational age (months), diabetes and gestational diabetes. The third module screened for alcohol use. Women that screened positive for alcohol, i.e., reported current use (in the past 30 days) or prior use (12 months before pregnancy) then completed the fourth and fifth modules. These modules assessed alcohol consumption in detail, including alcohol drink size, and delivered the alcohol intervention respectively.

Women who did not report alcohol use skipped out of the fourth and fifth module and were taken to the sixth module that screened for SSB use. Women reporting alcohol use also completed this sixth module after completing the fourth and fifth module as described above. Those reporting any SSB use in the past month completed the seventh and eighth modules on SSB drink size assessment and SSB brief intervention, respectively. To minimize user burden, only those reporting SSB use but not screening positive for alcohol use completed these two SSB modules. Thus, program users completed the assessment and intervention for only one beverage, alcohol or SSB, but not both beverages. All women then completed modules that screened for drug use and assessed user experience.

#### Alcohol screening

The alcohol use module began with a single question, asked in routine screening at the WIC clinic conducted by nutritionists, “When was the last time you drank alcohol?” Response options in the computerized program included “within the past month”, “within the past three months”, “3 to 6 months ago”, “6 to 12 months ago”, “more than 12 months ago”, and “I have never had alcohol”. Many women do not drink when pregnant; some may hesitate to report drinking during pregnancy. To ensure delivery of the intervention to all alcohol users, we additionally asked about alcohol use in the 12 months prior to pregnancy for all women who did not report recent (past month) drinking.

Women reporting alcohol use in the past month or prior to pregnancy were asked about frequency of alcohol use, types of alcoholic beverages consumed, and the two most frequently consumed beverages in each relevant time period. These details about drinking were assessed in two separate modules based on the time frame of most recent use. Thus, women who reported recent (past month) alcohol use were asked about their drinking in the past 30 days. Those not reporting current use and reporting drinking in the 12 months prior to their pregnancy were asked about their drinking in the year prior to their pregnancy. Women who reported drinking in a time frame that coincided with their pregnancy but denied both past month use and use in the year prior to their pregnancy (n = 4) were precluded from the detailed alcohol use assessment due to additional costs to program separate modules and the limited resources we had for software programming.

#### The alcohol drink size assessment

This module examined user’s beverage-specific drink (pour) sizes for the two most frequently consumed beverages by determining: a) the drink container or vessel that the participant usually drank from, and b) how much they would pour into their chosen vessel. Actual drinking vessels (9 glasses) were displayed in a box placed above the kiosk. If one of the 9 glasses was not the chosen drink container, photographs of other drink containers (glasses, bottles, cans) were presented on the computer screens to assess beverage-specific drink sizes. Women indicated their pour size in the container they selected by using letter markings on their chosen vessel. More details on the drink size assessment are available elsewhere [6].

#### The alcohol intervention

This module comprised two key components: feedback on drink size and a personalized plan for reducing consumption. The module began with individualized feedback on the woman’s drink sizes, reviewed discrepancies between her drink size and the standard size for each beverage, including that for usual and maximum consumption, and highlighted where pertinent, that she may be drinking more than she thinks she does. The module ended with a personalized plan for reducing consumption which included goal setting, an analysis of high risk situations for drinking alcohol, and suggested coping strategies.

Women who did not select complete abstinence (stop drinking) as a goal were encouraged to reduce consumption and to set a goal for reduced drinking. They were then guided through ways of doing so, such as to replace half the drinks per week with non-alcoholic drinks, drink one less each drink each week, or drink one less day each week. For women reporting current (past month) alcohol use, the intervention also provided information on the harmful effects of drinking during pregnancy. For women who did not report current use but use in the 12 months prior to pregnancy, information and feedback focused on reducing harms related to drinking alcohol in the future, should they choose to resume drinking.

#### Sugar sweetened beverage (SSB) screening

SSB screening included items on consumption of four types of sugar sweetened beverages in the past 30 days, including energy drinks, aqua fresca or horchata, regular soda or pop, and sweetened fruit drinks. Sugar-free or diet drinks were not included. Items used were from prior national surveys [19,20] with an additional fourth item on beverages popular with Hispanic women (e.g., agua fresca) and incorporated wording changes to the national survey items, such as descriptions and brand names for each beverage type (e.g., Kool-aid for sweetened fruit drinks).

#### SSB drink size assessment and intervention

These modules were completed by all women who screened positive for SSB use by reporting consumption of any of the four SSB types in the past month. The modules were identical in format to that for alcoholic beverages described previously and focused on the two most frequently consumed sugar sweetened beverages.

### Pilot implementation of the computerized SBI program

All women signing in for their first appointment at the clinic were provided with a handout describing the study along with other paperwork routinely given to women while checking in for their appointment. Women approached the computer kiosk voluntarily, selected the language they wanted to use and “continue” on the bilingual introductory screen to begin the program. To maximize participation in this new program, no identifying information, such as date of birth, medical record number, or phone number was obtained from program users.

### Study measures

#### Feasibility

This was measured by the time it took each user to complete the program. The program recorded the time, in minutes and seconds, at which the woman touched the “continue” button on the first screen to start the program and the time at which the last screen was reached. For those who did not complete the program, this last screen appeared upon their pressing “stop/discontinue”.

#### Acceptability

Program acceptability was assessed through items on user experience which included “Is this program a good idea for asking women about their drinking?”, “Was the computer program easy to use?”, “Did you learn anything new?”, “Will you talk about what you learned from the computer program with other people?”, “Will you talk with your doctor or clinic staff about anything you learned?”, “Did you feel you had enough privacy to answer the questions in this program honestly?”, and “Was it difficult to understand the questions or program materials?”.

#### Validity

To assess validity of the assessment of prenatal alcohol use by the program, we compared overall rates of alcohol use among pregnant women attending the WIC clinic with that based on responses to the alcohol use screening item in the computerized program. In order to determine whether a woman drank at a time when she was pregnant, WIC compares drinking data recorded by nutritionists in terms of calendar month and year with the number of months of pregnancy computed from the estimated delivery date in the medical record. We estimated alcohol use during pregnancy by cross-tabulating the woman’s response to the item on most recent alcohol use (“within the past month”, “within the past three months”, “3 to 6 months ago”, “6 to 12 months ago”, “more than 12 months ago”, and “I have never had alcohol”) by the number of months of pregnancy she reported in the program’s demographics module.

### Data analysis

To assess representativeness of our sample, we compared program user demographics with those of WIC clinic attendees during the months of data collection for the present study (June 2012 to January 2013). WIC maintains self-reported data obtained at the first clinic visit for all women enrolled in the program at the end of each month. Monthly data are not mutually exclusive and include several of the same women across months. To estimate demographic distributions for WIC attendees for the 7 months of our data collection, we averaged percentages for demographic groups in the WIC data (numbers per month ranged from 765 to 840) and compared these percentages to those for our sample using estimations of effect sizes to compare proportions [21].

Analyses assessing feasibility and acceptability examined time to complete the program and overall proportions of women endorsing different response options to the user experience items, respectively. Differences in feasibility and acceptability by user demographics and type of beverage (alcohol versus SSB) intervention received were also examined using T-tests (time to complete program) and chi-square tests (user-experience). Analyses assessing program validity used chi-square tests to compare the overall proportion of women identified as using alcohol during pregnancy by the computerized SBI program with monthly estimates in WIC data obtained via in-person screening by WIC nutritionists. We used the Statistical Package for Social Sciences (SPSS) version 15 for all analyses.

### Details of ethics approval

All study protocol and procedures were reviewed and approved by the Institutional Review Boards of the Public Health Institute (IRB# I11-014) and the Contra Costa Medical Research Center (no formal approval number).

## Results

### Participation and completion rates

A total of 450 women began the program and completed initial screens up to informed consent. Data from the informed consent screens indicated that 69% (310 of 450) of the women agreed to study participation (see Figure 1). Fourteen women were subsequently deemed ineligible for the study due to not being currently pregnant (n = 13) or using the program before (n = 1). Six additional women discontinued the program prior to the first beverage (alcohol) module and are not included in the analyses. Of the remaining 290 women, 263 (91%) completed both beverage screening modules and the pertinent assessment and intervention based on their beverage use. Program acceptability was examined for the 251 pregnant women (87%) who completed all the program modules.

### Descriptive characteristics of study participants

A majority of women using the program (see Table 1) were less than 30 years of age (67%), married or living with a partner (60%), had a high school or less education (62%) and were predominantly non-white (95%). Forty percent completed the computerized SBI program in Spanish.

Information on gestational age, clinic visits and diabetes obtained by the program, not reported in the tables, are detailed next. In order to keep the program as time efficient as possible, we did not assess several other pregnancy details, such as whether the pregnancy was the user’s first pregnancy and if they had other children. Half of all women were in their first trimester of pregnancy (n = 146), roughly a quarter were in their second (n = 77) and third (n = 67) trimester, respectively. Three-quarters of the women (72%) reported they received nutritional counseling at WIC, and 60% were receiving prenatal care outside the health center that the clinic was a part of. Twelve percent of women reported ever being told that they had diabetes. Of these women, 65% reported diabetes only during this pregnancy, 16% reported diabetes in both a prior and the current pregnancy.

### Representativeness of study participants compared to the WIC clinic

Small effect sizes (.10 or less) obtained indicated that our sample was representative in terms of age, language, marital status, and ethnicity. However, our sample had more women with more than a high school education (medium effect size, 38% vs 12%) and fewer women in their first trimester (medium effect size, 50% vs 69%, not shown in Table 1) compared to WIC clinic attendees. We note that WIC data regarding both education and trimester of pregnancy had a large percentage of missing values (28% and 24% respectively).

Clinic records indicate that an average of 140 new pregnant women enroll in the WIC program each month. Therefore, we calculated that 980 pregnant women visited the clinic for the first time over the seven months of data collection. Of the 263 pregnant women who completed the screening and brief intervention modules, most (but not all) were first-time visitors: 215 (82%) reported attending the clinic for the first time. Thus, we estimated that the reach of our SBI program for pregnant women visiting the clinic for the first time was 22% (215/980).

### Characteristics of women who reported using alcohol during pregnancy

Roughly twenty one percent (n = 60) of all women who completed the alcohol screening reported that they last used alcohol during a time period that coincided with their pregnancy (e.g., last use 3 months ago for a women who reported being 6 months pregnant). Compared to those who did not drink during pregnancy (see Table 1, column 3), prenatal alcohol users were more likely to be black, single, divorced, or separated and to have more than a high school education; and less likely to be Hispanic or use the Spanish version of the computerized SBI program. Detailed Information on alcohol use patterns assessed by our program is not reported here due to being outside the scope of the present paper. Consumption information is also limited by the small sample of drinkers.

### Past month SSB use

A large majority (87%) of women reported SSB use but did not differ in demographics from women who did not use SSB or from the general WIC population of pregnant women (see Table 1 column 4).

### Feasibility of the computerized SBI program

On average, most women completed the program in 10 to 15 minutes (see Table 2). Time to complete all modules, including the drug use and user experience module ranged from 3 to 31 minutes. When all women who completed the beverage use modules but not the complete program were included without regard to type of beverage intervention received (not shown in Table 2), time estimates averaged higher for the Spanish version took longer (mean = 14:36, SD = 5:17, n = 115) than the English version (mean = 9:05, SD = 3:54; T = 10.17, df = 287, p < .0001). This difference was also seen in the time needed to complete key beverage modules (see Table 2) in the Spanish versus English version for women who received the alcohol intervention (T = 8.78, df = 137, p < .0001) and for those who received the SSB intervention (T = 5.03, df = 122, p < .0001).

**Table 2:** **Mean time to complete computerized SBI program (Minute:Second) by modules completed, language chosen and beverage type**

	**Completed all program modules (n = 251)**							
	**Alcohol users^ (n = 136)**				**SSB users (n = 115)**			
	**N**	**Mean (SD)**	**Minimum**	**Maximum**	**N**	**Mean (SD)**	**Minimum**	**Maximum**
Overall	136	10:55 (4:43)	2:30	27:12	115	12.41 (5:39)	2:24	30.39
English language	93	8:59 (3:24)	2:30	19:04	54	10:15 (4:40)	2:24	26:43
Spanish language	43	15:05 (4:29)	8:03	27:12	61	14:50 (5:35)	4:21	30:39
Drank alcohol in past 30 days	5	14:40 (8:27)	3:12	27:12				
Drank alcohol in 12 months before pregnancy	131	10:46 (4:31)	2:30	23:14				
	**Completed beverage modules** ^**++**^ ** (n = 263)**							
	**Alcohol users (n = 139)**				**SSB users (n = 124)**			
	**N**	**Mean (SD)**	**Minimum**	**Maximum**	**N**	**Mean (SD)**	**Minimum**	**Maximum**
Overall	139	10:54 (4:41)	2:30	27:12	124	12:33 (5:43)	2:24	30:39
English language	96	9:01 (3:24)	2:30	19:04	57	10:00 (4:41)	2:24	26:43
Spanish language	43	15:05 (4:29)	8:03	27:12	67	14:43 (5:38)	3:04	30:39
Drank alcohol in past 30 days	5	14:40 (8:27)	13:31	27:12				
Drank alcohol in 12 months before pregnancy	134	10:45 (4:29)	2:30	23:14				

^**^**^Includes 15 women who consumed “just a sip” of the primary and/or secondary alcohol beverage.

^**++**^Includes program users who did not complete drug use or user experience modules that follow the beverage modules.

Overall, across both language versions, 90% of alcohol users completed the entire program in 17 minutes or less, 88% of the SSB users completed the program in 20 minutes or less (results not shown in table). When those completing the relevant assessment and intervention modules but not all program modules were included in program time estimates, 75% of alcohol users took 11 minutes or less, 80% of sweetened beverage users took 16 minutes or less.

### Acceptability of the program

Table 3 shows responses to the user experience items. Fifteen women who received the alcohol intervention reported consuming “just a sip” of the beverages they chose as their most frequently consumed alcoholic beverages. Because the intervention would be expected to have less salience for very light drinkers, we examined program acceptability separately for these women. The last column of Table 3 presents program acceptability data specific to these very light drinkers.

**Table 3:** **Acceptability of SBI program (user experience) by type of beverage use and intervention received (n = 251)**

	**Alcohol intervention (n = 15) %**	**SSB intervention (n = 115) %**	**Reported “just a sip” of alcohol, received alcohol intervention (n = 15) %**
Program was easy to use^&^	98	96	100
Learned something new	84	93*	80
Program a good idea for asking women about their drinking			
Prefer a person asking	15	13	0
Prefer the computer	30	23	33
Like either way	55	64	67
Will share the information learned from the program with others	79	85	87
Will talk with the doctor or WIC staff about anything learned from the program	68	84**	80
Had adequate privacy to answer the questions honestly	84	90	87
Difficult to understand questions or materials in the program?			
Yes, a lot	5	8	13
Yes, a little	4	10	0
No, not at all	90	82	87

*p < 0.05; **p < 0.01.

^&^Statistically significant differences between very high percentages are unlikely to be meaningful and were not assessed.

Overall (results not shown in Table), large majorities reported that the program was easy to use (97%), they learned something new (88%), thought the program was a good idea (27% preferred the computer, 60% said they liked either way (by person or computer), would share what they learned from the program with others (83%) and with their doctors or clinic staff (76%), and did not have any difficulty with the program materials (87%). Women receiving the SSB versus alcohol intervention were more likely to say that they learned something new and that they would talk to the doctor or WIC staff about what they learned. Acceptability did not differ between women reporting drinking only sips of alcohol and other alcohol users.

Because our sample was comprised of a significant proportion of women with more than a high school education, we also examined if education was associated with program acceptability (see Table 4). No differences were found by education on most items. However, users with less than high school education were more likely to report learning something new from the program.

**Table 4:** **Program user experience by demographics (n = 251)**

	**Education**		**Language**		**Race/Ethnicity**		**Age**	
	**< High school (n = 57)**	**High school or more (n = 194)**	**English (n = 147)**	**Spanish (n = 104)**	**Non-hispanic (n = 84)**	**Hispanic (n = 167)**	**18-25 (n = 118)**	**Older than 25 (n = 133)**
	**%**	**%**	**%**	**%**	**%**	**%**	**%**	**%**
Program was easy to use	98	97&	99	95	99	96&	98	97^&^
Learned something new	97	86*	82	97***	76	94***	88	88
Program a good idea to ask women about their drinking								
Prefer to talk to a person	7	15	16	9**	20	10	11	15
Prefer the computer	35	24	34	17	26	27	31	23
Like either way	60	61	50	74	54	63	58	62
Will share information learned from the program with others	90	80	75	93***	67	90***	83	82
Will to talk with the doctor or WIC staff about anything learned from the program	83	74	68	87**	63	82**	76	75
Had adequate privacy to answer the questions honestly	93	86	82	94*	83	89	86	89
Difficult to understand materials								
Yes, a lot	11	6	5	10	5	8	8	7
Yes, a little	5	7	5	9	6	8	8	5
No, not at all	84	87	90	81	92	84	84	88

*p < .05, **p < 0.01; ***p < 0.001.

^&^Statistically significant differences between very high percentages are unlikely to be meaningful and were not assessed.

Comparison of program acceptability by other demographics, including language, race/ethnicity and age, indicated that compared to women using the English version and non-Hispanic women respectively, those using the Spanish version and Hispanic women were more likely to agree that they learned something new, more likely to share the information with others, talk to the doctor or staff about what they learned from the program, and report adequate privacy to answer questions, and less likely to prefer a person ask about their drinking. No differences were found in program acceptability by age.

Finally, 32 of the 251 women completing the user experience items responded “no” (n = 24) or “not sure” (n = 8) to “Did you feel you had enough privacy to answer the questions honestly?’. Therefore we also examined program acceptability by reporting of privacy (results not shown in tables). These 32 women were less likely to report that they learned something new or share program information with others or with their doctor, more likely to prefer a person asking about their drinking, and completed the program in less time than those who reported adequate privacy.

#### Validity of screening for prenatal alcohol use

The prenatal alcohol use rate obtained by our program was 20.6% (60 of 290). This rate was significantly higher than the highest rate of 15.2% (χ^2^ = 6.78, p < .01) and the average rate of 13.3% (χ^2^ = 13.73, p < .001) in the WIC data for the same time period.

## Discussion

### Main findings

This study demonstrated high feasibility and acceptability of a new, computerized Screening and Brief Intervention (SBI) program for alcohol and sugar sweetened beverage (SSB) consumption among pregnant women in a public health clinic. Almost all women completed the program in 15 minutes or less and none required staff assistance. Regardless of the type and language of intervention received, women also responded very positively to the program and found the program information novel and worth sharing with others, including their doctor and health care professionals. Finally, our study documented higher identification rates for prenatal alcohol use than that obtained by routine clinic screening. Although the higher rates of alcohol use may be due to our sample having women with more education and educated women being more likely to drink alcohol, we note that the clinic data was missing information on education for roughly one in four women. Thus any biases in our findings due to our specific sample may be artificially inflated due to missing data in the clinic records.

While the 67% study participation rate may appear relatively low, it is at best a conservative estimate of future use of computerized SBI given the lack of active recruitment and incentives in our study. Since the kiosk was a stand-alone, un-monitored tool, ineligible users (non-pregnant women, children, spouses) may have declined participation and, thus, inflated the study refusal rate. We estimated the reach of our program for pregnant women visiting the clinic for the first time to be 22%. It is noteworthy that 18% of our program users were non first time visitors who did not receive information about the kiosk program when they checked in at the clinic. This suggests that these women found the kiosk and program appealing and that future studies can enhance program reach by including all pregnant women attending the clinic and not just first time visitors. Future use of the program could also be increased by its integration into routine care, e.g., by having reception staff point out the kiosk to pregnant women checking in to the clinic, explain its short duration and ease of use, and clarify that women can complete the program while waiting or after meeting with clinic staff.

### Strengths

Our SBI includes several improvements over other SBIs. First, it extends SBI to another health risk behavior for weight gain and diabetes related obstetric complications [17], SSB consumption. Program users receiving the SBI for SSB endorsed its innovativeness, and were more likely than those receiving the alcohol SBI to report that they learned something new and would share the information with others, including their doctor. Given that heavy drinkers consume larger-than-standard alcoholic drinks, [8,9], and larger SSB drinks contain more sugar, the focus on drink-size assessment and feedback provides an creative approach to reducing harms related to SSB use, particularly where complete abstinence is not possible.

Drinking prior to pregnancy recognition is often missed when past month use alone is assessed [22,23]. Our program assessed both recent and prior alcohol use and intervened on future alcohol consumption in those reporting prior use and recent abstinence. Thus, it has the potential for reducing drinking prior to subsequent pregnancies. As 21% to 43% of women use alcohol prior to pregnancy recognition [22,24], intervening on future drinking is important.

A key innovation of our computerized SBI is that it is bilingual. As the fastest growing ethnic group in the United States [25] with the highest birth rates [14,26], Hispanics are expected to increasingly represent more women in public health clinics. Recent convergence of heavy drinking rates among White and Hispanic women of childbearing age [27] substantiates the need for computerized SBI in Spanish. Only a handful of prior studies have included Hispanic women [13,28], and most exclude women who do not speak English.

Embarrassment and fear of judgment can impede identification of prenatal alcohol use via face-to-face screening [29]. WIC staff routinely screen for alcohol use in person. When used as an anonymous, self-administered tool as in our study, computerized SBI may help overcome barriers to the identification of alcohol use that are inherent in face-to-face situations, such as staff concerns regarding discussing a sensitive behavior like drinking, and respondent stigmatization concerns related to disclosure of alcohol use. Women may be more willing to accurately report drinking to a computer than a person. Indeed, computerized SBI has been reported to be less threatening to hazardous drinkers than a face-to-face intervention [3].

Due to lack of data in WIC records on the frequency and quantity of alcohol consumption, particularly at the level of detail as in our program, we could not assess validity of alcohol use patterns. However, we expect that women will be more willing to more honestly report details about their alcohol use in a computer program than to a staff person. We also did not specifically assess validity for SSB use as it is not currently assessed by clinic staff but computerized programs may also minimize barriers to disclosure of SSB use. Overall, though, the higher identification rates obtained by our program for alcohol use support the use of computerized SBI in prenatal clinics.

### Limitations

Because the computerized SBI was completely anonymous, we lacked identifying information needed to compare alcohol use in SBI versus WIC staff reports at an individual level. Future studies should link data obtained by computer versus clinic staff to assess program validity.

Placing the computer kiosk in the clinic waiting area maximized its visibility and facilitated use by women but also reduced privacy. Women reporting lack of adequate privacy completed the program more quickly, and were less satisfied with it than those reporting adequate privacy. Use of a privacy screen for the monitor can interfere with use of the touch screen. However, future implementation can increase privacy with the use of a barrier, e.g., a wall, enclosure, or booth.

The self-selected nature of our program users and possible biases due to their higher education may also limit generalizability of our study findings. Finally, our study demonstrated feasibility and acceptability but not efficacy of computerized SBI because of lack of follow up outcome data. Examining program efficacy in reducing alcohol and SSB consumption and improving maternal and child outcomes with controlled studies is a key area of focus for our planned research.

## Conclusions

Our findings suggest that computerized SBI provides a low resource, time-efficient, acceptable and valid tool to address gaps in healthcare for women aimed at preventing poor birth and fetal outcomes. Low-income women are less likely to receive appropriate counseling regarding alcohol use during pregnancy [30,31] and to our knowledge, there is no SSB intervention for pregnant women. Bilingual, computerized SBI in public health clinics can enhance prevention efforts where they are most needed. Future research critically evaluating computerized SBI efficacy in reducing alcohol and SSB consumption is needed to build an evidence-base for its use.
