# Erratum: Feasibility and acceptability of a novel, computerized screening and brief intervention (SBI) for alcohol and sweetened beverage use in pregnancy

**DOI:** 10.1186/s12884-015-0582-4

**Published:** 2015-08-18

**Authors:** Madhabika B. Nayak, Rachael A. Korcha, Lee A. Kaskutas, Lyndsay A. Avalos

**Affiliations:** Alcohol Research Group-Public Health Institute (ARG-PHI), 6475 Christie Avenue, Suite 400, Emeryville, CA USA; ARG-PHI & School of Public Health, University of California, Berkeley, USA; Kaiser Permanente Northern California, Division of Research, Oakland, CA USA

## Erratum

After publication of the original article [1] it came to the publishers attention that one name had been inadvertently misspelt. The correct authors’ list should read as indicated above.

We apologize for any inconvenience this has caused.

## References

[1] Nayak MB, Korcha RA, Kaskustas LA, Avalos LA (2014). Feasibility and acceptability of a novel, computerized screening and brief intervention (SBI) for alcohol and sweetened beverage use in pregnancy. BMC Pregnancy and Childbirth.

